# Improving understanding of carbon stock characteristics of *Eucalyptus* and *Acacia* trees in southern China through litter layer and woody debris

**DOI:** 10.1038/s41598-020-61476-3

**Published:** 2020-03-13

**Authors:** Hui Zhang, Yinhua Jiang, Mingwei Song, Jiajun He, Dongsheng Guan

**Affiliations:** 1 School of Chemistry and Environmental Engineering, Wuhan Institute of Technology, 430205 Wuhan, China; 2 School of Management, Wuhan Institute of Technology, 430205 Wuhan, China; 3 College of Resources and Environment, Huazhong Agricultural University, 430070 Wuhan, China; 4 School of Environmental Science and Engineering, Sun Yat-sen University, 510275 Guangzhou, China

**Keywords:** Carbon cycle, Forestry

## Abstract

Surveying the quality and quantity of carbon stock in litter layer and woody debris of *Eucalyptus* and *Acacia* plantations is critical in understanding their carbon pools. Here, the focus of the present study was on a number of *Eucalyptus* and *Acacia* plantations of different stand aged in the Pearl River Delta region of southern China. The plantation type proved to be a crucial driver of the carbon concentration in litter layer and woody debris, with *Acacia* exhibiting a superior ability to *Eucalyptus* to accumulate carbon with stand age in both these materials. The relative contribution of the litter layer and woody debris to the carbon stock of the ecosystem was also significantly higher under mature *Acacia* (8% and 7%, respectively) than that under mature *Eucalyptus* (4% and 1%, respectively). Most of the carbon stock within the litter layer was present in the leaf debris. The carbon stock in woody debris was mainly contained in the components within the 10–20 cm diameter class during the primary decay stage, represented as snags in middle-aged and mature *Acacia*, and as logs for mature *Eucalyptus*, respectively. The results indicate that both plantation type and stand age influence the characteristics of carbon stored in litter layer and woody debris significantly.

## Introduction

The litter layer and woody debris constitute important components of forest ecosystem, and are accumulated on the forest floor, serving as a source of organic matter and nutrients to the soil^1–3^. Assessment of the quality and quantity of the woody debris has led to the discovery that its quantity, type, diameter class and decay class are determined by stand structure, ecosystem function and forest management history^1–4^. The management of the forest ecosystem is increasingly viewed as a way of mitigating against the negative effects of climate change^5,6^. The stock of carbon (C) stored in litter layer and woody debris, along with its contribution to the ecosystem, is assumed to depend largely on the tree type and age^7–9^. Generally, in natural/secondary forests, a substantial proportion of C stock is contained in litter layer and woody debris, e.g., in a secondary forest aged 75 years, they contain an estimated 20% of the total ecosystem accumulation of C stock^10^. In subtropical evergreen broad-leaved forests, the relatively low turnover rate of woody debris (<22 year) led to a relatively slow release of C^2^, indicating that woody debris is an important component of long-term C pools in these areas. Most attempts to characterize the C stock in the woody debris of forests growing in subtropical climate zone have focused on natural/secondary evergreen broad-leaved forests^2,8^. However, the relationship of the quality and quantity of woody debris C stock with tree species and ages in subtropical plantations remain unclear. Currently, China contains the largest land mass of plantations in the world (~69 million ha in 2013)^11^. An estimated 80% of the increase in the national C sink can be attributed to forest plantations, with those in southern China representing 65% of the national C sink^12^. *Eucalyptus* spp. and *Acacia* spp. are tree species native to the tropical zone. Owing to their rapid growth rate and ability to thrive on poor soil, both *Eucalyptus* (non-N_2_-fixing species) and *Acacia* (N_2_-fixing species) have been planted widely in the Pearl River Delta (PRD) region of southern China during a reforestation initiative of the provincial government of Guangdong, targeting areas where the soil had become badly degraded in the 1980s.

The relative effectiveness of these species to sequester C is in question. Certain studies have suggested that the size of the soil C pool does not respond to afforestation with *Eucalyptus* and *Acacia* trees^13,14^, while others have reported the contrary results, claiming that the C sequestration ability of *Acacia* is generally superior to that of *Eucalyptus*^15^. A thorough study on the quality and quantity of C stock in litter layer and woody debris is critical in understanding the role of the C-sequestration capacity of N_2-_fixing and non-N_2_-fixing trees and their difference. However, insufficient data exist on the characteristic of the C source, including the C stock in the forest floor layer, for these two plantation types. Typically, *Eucalyptus* and *Acacia* plantations used for wood production are maintained for under ten years^16,17^. To the best of our knowledge, no extensive study has been performed to date on the long-term dynamic characteristics of the C stock in the litter layer and woody debris and the association with tissue type and stand ages for *Eucalyptus* and *Acacia* plantations in subtropical zone. The quantity of stored C is dependent on both the volume of the biomass and the relative C concentration therein. The C concentration in the litter layer and the woody debris is rarely measured directly; rather, there is a tendency to assume a value of 50%^18,19^.

However, an increasing amount of evidence now indicates that this proportion depends on various parameters, including tree species, stand age, stand condition, local climate, decay stage/decay class and tissue type^2,6,20–22^. For example, certain studies have reported that the C concentration of litter and woody debris not only varies significantly by forests types^20,23^, but also that it is age-dependent in natural forests as well as plantations^2,6^. Given the wide range of C concentration in the litter layer of subtropical forests (40–58%)^6,7,23,24^, and in the woody debris in tropical and subtropical forests (20–52% and 45–61%, respectively)^22,25^, this value should be measured in order to improve the accuracy of C stock estimation. The present study therefore aimed to characterize the C pools stored in samples of the litter layer and woody debris of 21 *Eucalyptus* and *Acacia* plantations in the PRD region. Our specific objectives were to test the three hypotheses: firstly, that the C concentration of the litter layer and woody debris is dependent on either the species of tree being grown and/or the stand age; secondly, that forest type and stand age affect the C stock held in the woody debris of a specific diameter class and/or state of decay; and finally, that both forest type and stand age contribute to the capacity of a plantation to store C in the litter layer and/or the woody debris.

## Materials and methods

### Site description

The PRD region lies in Guangdong Province, China (21°31′–23°10′N, 112°45′–113°50′E). Its climate is characterized by hot, humid summers and mild winters. A large proportion of the mean annual rainfall of 1,600 mm falls during the months of July and August. The mean annual temperature is approximately 21 °C, with daytime temperatures exceeding 30°C on around 120 days per year. In the present study, a set of 21 plantations was studied: ten were planted exclusively to *Acacia* and 11 exclusively to *Eucalyptus*.

The two types of rapidly growing trees species were planted in degraded red soil, on hilly sites in the neighborhood of the cities of Heshan, Zhuhai and Guangzhou. The monitoring period was November 2010 to January 2011. The stand age of the plantations was classified as either young (<6 years), middle-aged (6–15 years) or mature (>16 years). The mortality rate of canopy in young *Acacia* plantations was 5.3 ± 1.2%, while there was no appreciable mortality in the young *Eucalyptus* plantations. The mortality rates in the middle-aged plantations were 11.9 ± 5.0% for *Acacia* and 4.4 ± 1.2% for *Eucalyptus*; in the mature plantations, the respective rates were 14.9 ± 6.5% and 4.0 ± 1.7%. Further descriptive details of the sites are given by Zhang *et al*.^26^ and are reproduced in Table 1.

**Table 1:** The basic characteristics in the experimental forest stands.

No.	Latitude	Longitude	Constructive Species	Stand age (a)	Mean DBH (cm)	Mean tree height (m)	Stand density (trees·hm^−2^)
1	N.23°11′	E.113°22′	*E. citriodora*	18.0	17.8 ± 13.1	19.4 ± 6.8	933.3
2	N.23°11′	E.113°22′	*E. urophylla*	4.0	8.7 ± 4.0	12.0 ± 3.9	2777.8
3	N.23°18′	E.113°23′	*E. urophylla*	16.0	15.2 ± 15.3	16.9 ± 4.6	1344.4
4	N.22°57′	E.113°18′	*E. urophylla*	8.0	12.3 ± 5.9	14.9 ± 3.4	1600.0
5	N.23°18′	E.113°25′	*E. urophylla*	11.0	13.6 ± 4.2	14.8 ± 3.9	1744.4
6	N.22°21′	E.113°34′	*E. urophylla*	1.5	6.1 ± 3.2	7.1 ± 2.6	2866.7
7	N.23°11′	E.113°23′	*E. urophylla*	2.5	7.8 ± 4.0	10.0 ± 3.9	2622.2
8	N.22°21′	E.113°34′	*E. urophylla*	6.0	11.3 ± 4.9	14.7 ± 3.0	1653.3
9	N.22°40′	E.112°54′	*E. urophylla*	1.0	5.9 ± 2.5	5.1 ± 2.9	2777.8
10	N.22°40′	E.113°54′	*E. urophylla*	25.0	12.2 ± 4.7	20.3 ± 3.1	1822.2
11	N.22°40′	E.112°54′	*E. urophylla*	10.0	12.3 ± 4.9	14.2 ± 3.5	1877.8
12	N.23°40′	E.113°25′	*A. mangium*	12.0	12.6 ± 8.4	9.7 ± 4.2	1111.1
13	N.23°18′	E.113°25′	*A. mangium*	17.0	17.8 ± 8.9	13.6 ± 3.9	816.7
14	N.23°18′	E.113°23′	*A. mangium*	5.5	9.1 ± 7.8	7.1 ± 2.0	1544.4
15	N.23°18′	E.113°23′	*A. mangium*	4.5	8.9 ± 4.4	7.0 ± 2.4	1555.6
16	N.22°21′	E.113°34′	*A. auriculaeformis*	15.0	14.7 ± 7.8	11.9 ± 4.6	1200.0
17	N.22°21′	E.113°34′	*A. auriculaeformis*	13.0	12.2 ± 5.0	9.5 ± 3.7	1222.2
18	N.22°20′	E.113°34′	*A. auriculaeformis*	8.0	10.2 ± 4.7	8.2 ± 2.7	1444.4
19	N.22°40′	E.112°53′	*A. mangium*	26.0	20.7 ± 10.2	16.1 ± 4.4	833.3
20	N.22°40′	E.112°54′	*A. mangium*	5.0	8.1 ± 2.8	7.1 ± 1.4	1522.2
21	N.22°41′	E.112°53′	*A. mangium*	18.0	17.7 ± 6.2	15.0 ± 2.2	840.0

Note: Values given as mean ± SD.

### Sampling

Each plantation was sampled by a single 30 m × 30 m square, in which the tree heights above 3 m were measured using a digital hypsometer, while their diameter at breast height (1.3 m above ground level) (hereafter DBH) was obtained using a diameter tape. Allometric equations suggested by Ye *et al*.^27^ and Ren *et al*.^28^ were subsequently used to calculate the biomass of the arbor layer. The understory biomass was estimated by destructive sampling from the central 1 m^2^ of five 2 m × 2 m plots, including all shrubs, ferns and grasses. The shrubs were separated into twigs, branches, leaves and roots, and the herbaceous species into above- and below-ground material. After obtaining fresh weights, a weighed sub-sample was oven-dried at 65 °C until constant weight (dry weight), and the loss of weight was used to estimate tissue water content. The litter layer contribution was obtained from a set of five 1 m × 1 m quadrats arranged along the diagonal of the 30 m × 30 m square: it comprised leaf debris, fallen fruits, bark and fallen twigs (<10 mm diameter). The fresh weight of each component was measured on site and its dry weight was measured after oven-drying. Following the suggestions of Wei *et al*.^29^ and Tang and Zhou^30^, woody debris measurements were restricted to snags (standing dead trees), logs (fallen dead trees) of minimum end diameter 2.5 cm and minimum length 1 m. The woody debris was organized into four diameter-based classes: 2.5–10 cm, 10–20 cm, 20–30 cm and >30 cm. The selected woody debris pieces were divided into five decay classes using a five-step wood decay classification: classes I and II reflect an early stage of decay, classes III and IV an intermediate stage, and class V an advanced stage. To determine wood density, we cut small pieces of wood from woody debris based on decay class and species. In total, we obtained approximately 25–30 fragments for each plot. Moisture contents were obtained by oven-drying, while their volume was inferred using a water displacement method.

### Laboratory analysis

The quantity of C present in vegetable tissues was calculated by multiplying biomass by C concentration. Total ecosystem biomass was the sum of the dry weight stored in these four vegetable tissues, including the canopy, understory layers, litter layer and woody debris. Tissue C concentration was determined via digestion in acidified potassium dichromate (LY/T1237–1999, Forestry Standards of the People’s Republic of China). A 0.0050–1.0000 g sample was digested in 5 mL 0.8000 M K_2_Cr_2_O_7_ plus 5 mL 18.4 M H_2_SO_4_, then heated to 170–180 °C for 5 min. The resulting solution was titrated against 0.2 M FeSO_4_.7H_2_O. In the litter layer and woody debris, the overall C stock in the original sample was obtained by multiplying the C concentration by the sample weight. The calculated biomass in the canopy and understorey layers were converted into fixed C by multiplying by 0.5^31^.

### Statistical analysis

The mean C concentration and C stock, and respective associated standard deviation (SD) of the litter layer and the woody debris from young, middle-aged and mature *Eucalyptus* and *Acacia* plantations were calculated. A one-way analysis of variance (ANOVA) was used to test for any effect of forest type and/or stand age on either the proportion or quantity of C stored in the litter layer and woody debris, also including respective various components, woody debris at different diameter class and decay classes. A Tukey’s HSD test was used for comparison of means. All calculations and statistical analyses were carried out by Microsoft Excel 2016 and IBM SPSS (Version 22.0, SPSS Inc., USA).

## Results

### C concentration of litter layer and woody debris

There was a significant effect of forest type on the C concentration of both the litter layer and the woody debris, independent of stand age. In the *Eucalyptus* plantations, the mean C concentration was 44.5% (litter layer) and 45.8% (woody debris), while in the *Acacia* plantations, the equivalents were 48.3% and 48.9%, respectively (Table 2). Within the litter layer, the C concentration of the leaf and branch material was significantly (*P* < 0.01) higher in the *Acacia* material (47.6–49.3% & 46.4–49.6%) compared with the *Eucalyptus* leaf (44.2–47.3%) and branch (42.8–44.2%) materials. Regarding the woody debris, the C concentration of snags and logs was significantly (*P* < 0.05) higher in the *Acacia* (47.4–51.0% & 47.6–48.0%, respectively) than in the *Eucalyptus* (46.5–47.1% & 45.7–44.8%, respectively) plantations.

**Table 2:** The C concentration in the various component of litter layer and woody debris for *Acacia* and *Eucalyptus* plantations (%).

Type	Period	Leaf	Brach	Bark	Fruit	Litter layer	Snags	logs	woody debris
*Eucalyptus*	Young	46.13 ± 0.89	44.22 ± 3.86	41.45 ± 1.24	42.20 ± 4.06	45.03 ± 1.54	—	—	—
	Middle-aged	44.18 ± 1.82	42.91 ± 2.00	39.91 ± 2.09	43.31 ± 2.38	43.26 ± 1.78	46.54 ± 2.92	45.72 ± 1.68	46.02 ± 2.23
	Mature	47.29 ± 3.00	42.82 ± 1.61	43.05 ± 2.22	40.59 ± 0.69	45.27 ± 1.86	47.11 ± 1.10	44.80 ± 0.83	45.48 ± 1.07
*Acacia*	Young	47.58 ± 0.91	46.36 ± 1.38	43.31 ± 0.56	44.11 ± 1.22	47.25 ± 0.56	47.40 ± 1.92	47.55 ± 1.30	47.52 ± 1.37
	Middle-aged	49.33 ± 3.30	49.60 ± 2.58	42.79 ± 1.71	45.32 ± 1.50	49.09 ± 2.67	50.25 ± 2.13	46.87 ± 1.83	49.30 ± 1.58
	Mature	49.21 ± 2.28	46.91 ± 4.04	42.36 ± 0.87	43.44 ± 2.84	48.60 ± 2.61	51.03 ± 1.56	47.95 ± 1.20	49.96 ± 0.84
Forest types		**	**	ns	*	**	*	*	**

Note: — means no statistic data, ns denotes not significant, while * and ** represent significant differences at *P* < 0.05 level and extremely significant difference at *P* <0.01 level between the *Eucalyptus* and *Acacia* plantations, respectively, same as blow.

### Carbon stock of the litter layer and the woody debris

#### Litter layer

The C stock of the litter layer, and the leaf and branch material contained within it increased substantially as stand age (Tables 3 and 4). Leaf debris and fallen branches dominated the C stock of the litter layer (87.3% in *Eucalyptus* plantations and 95.7% in *Acacia* plantations). The leaf material had the largest contribution to the C stock of this layer (73% under *Eucalyptus* and 53% under *Acacia*), which differed significantly (*P* < 0.01) between the two forest types: it was higher in the *Acacia* plantations than in the *Eucalyptus* ones.

**Table 3:** The C stored in the various component of litter layer and woody debris of *Eucalyptus* and *Acacia* plantations (t·ha^−1^).

Type	Period	Leaf	Brach	Bark	Fruit	Litter layer	Snags	logs	WD	C stock of ecosystem
*Eucalyptus*	Young	0.49 ± 0.14Aa	0.29 ± 0.26a	0.05 ± 0.04a	0.08 ± 0.07a	0.91 ± 0.40A	—	—	—	29.75 ± 17.42a
	Middle-aged	1.37 ± 0.43a	1.26 ± 0.75b	0.22 ± 0.11ab	0.12 ± 0.08a	2.97 ± 0.66Ba	0.40 ± 0.20	0.18 ± 0.14	0.58 ± 0.23	75.67 ± 12.44b
	Mature	2.73 ± 1.02Bb	1.07 ± 0.20ab	0.39 ± 0.32b	0.16 ± 0.14a	4.34 ± 0.63Bb	0.46 ± 0.36	0.75 ± 0.06	1.21 ± 0.30	103.16 ± 3.69c
*Acacia*	Young	1.55 ± 0.09Aa	0.28 ± 0.06Aa	0.05 ± 0.01a	0.02 ± 0.02a	1.91 ± 0.17A	0.10 ± 0.05A	0.33 ± 0.10a	0.43 ± 0.13A	43.84 ± 2.70a
	Middle-aged	3.42 ± 0.78Ab	2.04 ± 0.48Bb	0.15 ± 0.09ab	0.11 ± 0.05b	5.73 ± 1.12Ba	2.76 ± 1.04Ba	1.80 ± 1.33ab	4.56 ± 1.11B	70.53 ± 13.02b
	Mature	5.83 ± 1.21B	1.43 ± 0.68Ab	0.22 ± 0.06b	0.09 ± 0.07ab	7.57 ± 0.95Bb	3.94 ± 1.01Ba	2.74 ± 1.30b	6.68 ± 0.38C	94.47 ± 11.95c
Forest types		**	ns	ns	ns	*	**	**	**	ns

Note: Values are shown as mean ± SD; different lower case (*P* < 0.05) and upper case (*P* < 0.01) letters within a column indicate a significant difference between the various ages of a given forest type; for instance, for C stored in leaf in *Acacia* plantations, the only significant difference (*P* < 0.05) was presented between young plantations and middle-aged plantations, while an extremely difference (*P* < 0.01) was observed between young plantations and mature plantations; ns: not significant, *significant difference (*P* < 0.05), **extremely significant difference (*P* < 0.01) between the *Eucalyptus* and *Acacia* plantations. The C stock of the ecosystem was obtained by multiplying the mass of each component by its C concentration. Thus, the ecosystem’s C stock represents the sum of the C stock of the canopy layer, the understory layer, the litter layer and the woody debris.

**Table 4:** C stored in the woody debris classified by the various diameter class in *Acacia* and *Eucalyptus* plantations of different stand age (t·ha^−1^).

Type	Period	2.5~10 cm			10~20 cm			20~30 cm			>30 cm		
		Snags	Logs	Total	Snags	Logs	Total	Snags	Logs	Total	Snags	Logs	Total
*Eucalyptus*	Young	0a	0Aa	0Aa	0a	0Aa	0A	0a	0Aa	0Aa	0a	0a	0a
	Middle-aged	0.117 ± 0.11b	0.050 ± 0.11Bb	0.167 ± 0.15ABb	0.199 ± 0.11b	0.116 ± 0.09Ab	0.314 ± 0.12Ba	0.085 ± 0.09a	0.014 ± 0.01Aa	0.098 ± 0.09ABa	0.00 ± 0.00a	0.00 ± 0.00a	0.00 ± 0.00a
	Mature	0.051 ± 0.01ab	0.158 ± 0.05Bc	0.210 ± 0.03Bb	0.202 ± 0.13b	0.405 ± 0.07Bc	0.606 ± 0.13Bb	0.157 ± 0.17a	0.124 ± 0.08Bb	0.280 ± 0.12Bb	0.047 ± 0.05b	0.064 ± 0.06b	0.111 ± 0.10b
*Acacia*	Young	0.044 ± 0.04a	0.213 ± 0.12a	0.256 ± 0.15a	0.056 ± 0.03a	0.117 ± 0.04a	0.174 ± 0.07Aa	0a	0a	0Aa	0a	0a	0a
	Middle-aged	0.210 ± 0.15a	1.232 ± 1.44a	1.442 ± 1.35a	1.183 ± 0.87ab	0.334 ± 0.15a	1.518 ± 0.73ab	0.813 ± 0.99ab	0.220 ± 0.39a	1.033 ± 0.96ab	0.556 ± 0.69a	0.012 ± 0.02a	0.568 ± 0.69a
	Mature	0.125 ± 0.06a	0.941 ± 0.43a	1.067 ± 0.45a	1.994 ± 1.04b	1.069 ± 1.46a	3.063 ± 1.52Bb	1.420 ± 0.58b	0.500 ± 0.06a	1.920 ± 0.62Bb	0.407 ± 0.31a	0.223 ± 0.39a	0.630 ± 0.40a
Forest types		ns	*	**	**	ns	**	*	*	**	*	ns	*

Note: Values are shown as mean ± SD; different lower case (*P* < 0.05) and upper case (*P* < 0.01) letters within a column indicate a significant difference between the various ages of a given forest type; ns: not significant, *significant difference (*P* < 0.05), **extremely significant difference (*P* < 0.01) between the *Eucalyptus* and *Acacia* plantations.

#### Woody debris

The C stock of woody debris was significantly (*P* < 0.01) higher in *Acacia* than that in *Eucalyptus* plantations (Table 3). The C stored in woody debris was strongly related to stand age, as was the individual contribution of snags and logs. The largest part of the C stock of woody debris was contained in the snags, especially in the middle-aged and mature plantations (Table 3). Material of diameter 2.5–10 cm contributed the most to the C stock, especially in young *Acacia* plantations (0.26 t·ha^−1^), which harbored about 60% of the total C stock in woody debris, mainly as logs (mean 75%) (Table 4). Material of diameter 10–20 cm contributed most strongly to the C stored in woody debris in the middle-aged and mature plantations, particularly for middle-aged *Eucalyptus* (54%) and *Acacia* plantations (46%). The quantity of C stored in the component was, respectively, 0.61 and 0.17 t·ha^−1^ in the middle-aged and mature *Eucalyptus* plantations and 1.52 and 3.06 t·ha^−1^ in the middle-aged and mature *Acacia* plantations. There was no statistical difference between the contributions made to the C stock by material of diameter >30 cm, irrespective of stand age.

Both plantation type and stand age exerted a strong influence over the quantity of C present in the various woody debris decay state classes (Tables 5 and 6): there was a significantly different representation of the primary (I and II) and intermediate (III and IV) classes both between the two plantation types and between the three stand ages. Most of the woody debris was present in decay class I. Overall, the primary decay stage in both plantation types contributed most strongly, especially in the *Eucalyptus* plantations. Thus, in the middle-aged and mature plantations, the mean contribution to the C stored in the woody debris from material at the primary stage of decay was, 93% (0.54 t·ha^−1^) and 69% (0.83 t·ha^−1^) for *Eucalyptus* plantations, and 76% (3.48 t·ha^−1^) and 57% (3.80 t·ha^−1^) for *Acacia* plantations, respectively. By contrast, material at the intermediate stage of decay contributed rather little: class IV material stored between 1% and 8% of the C stock. Notably, the woody debris in primary decay classes mainly presented in the form of logs and snags in both *Eucalyptus* and *Acacia* plantations.

**Table 5:** Characteristics of woody debris at different decay classes in the forest ecosystem.

Item	Primary		Intermediate		Advanced
	I	II	III	IV	V
Leaf	Present	Disappear	Disappear	Disappear	Disappear
Bark	Intact, tight	Intact on the whole, tight	Partly present, loose	Trace present, loose	Disappear
Branch	Twigs less than 3cm present, Branches keep intact	Twigs less than 3cm partly present, Branches keep intact	Absent of twigs, Branches present, but mostly broken	Absent of twigs, Branches partly present	Disappear
Wood consistency and color	Solid, original color	Solid, original color	Semi-solid, faded	Party solid, breakable	Soft, powdery
Moss or fungi	Disappear	Cover less than 25% of surface area	Cover 25%~50% of surface area	Cover more than 50% of surface area	Cover more than 50% of surface area
Root invading	Disappear	Disappear	Disappear	In sapwood	In heartwood

Note: Data is from Tang and Zhou^30^.

**Table 6:** The C stock in woody debris across various decay classes and plantation ages (t·ha^−1^).

Item		Primary						Intermediate					
		I			II			III			IV		
		Snags	logs	woody debris	Snags	logs	woody debris	Snags	logs	woody debris	Snags	logs	woody debris
*Eucalyptus*	Young	0Aa	0Aa	0Aa	0a	0Aa	0A	0a	0Aa	0Aa	0a	0a	0a
	Middle-aged	0.199 ± 0.10Bb	0.064 ± 0.05AB	0.263 ± 0.10Bb	0.177 ± 0.09b	0.100 ± 0.08Aa	0.277 ± 0.89B	0.024 ± 0.03ab	0.016 ± 0.03Aa	0.040 ± 0.05Aa	0a	0a	0a
	Mature	0.142 ± 0.10Aa	0.195 ± 0.05Bc	0.337 ± 0.14Bb	0.191 ± 0.13b	0.301 ± 0.08Bb	0.491 ± 0.04C	0.124 ± 0.13b	0.227 ± 0.07Bb	0.351 ± 0.09Bb	0a	0.028 ± 0.05a	0.028 ± 0.05a
*Acacia*	Young	0.057 ± 0.03Aa	0.124 ± 0.01a	0.181 ± 0.04Aa	0.043 ± 0.02Aa	0.206 ± 0.09a	0.249 ± 0.10a	0a	0a	0Aa	0a	0a	0a
	Middle-aged	1.207 ± 0.56Bb	0.693 ± 0.49a	1.901 ± 0.65Bb	1.032 ± 0.43ab	0.542 ± 0.42a	1.575 ± 0.45ab	0.523 ± 0.74a	0.523 ± 0.58ab	1.046 ± 0.87ABa	0a	0.039 ± 0.08a	0.039 ± 0.08a
	Mature	1.01 ± 0.37ABb	0.346 ± 0.30a	1.358 ± 0.65ABb	2.084 ± 1.11Bb	0.354 ± 0.37a	2.44 ± 1.48b	0.803 ± 0.40a	1.500 ± 1.14b	2.303 ± 0.74Bb	0.047 ± 0.08a	0.533 ± 0.50b	0.580 ± 0.57a
Forest types		**	*	**	**	*	**	*	*	**	ns	ns	ns

Note: Values shown as mean ± SD; different lower case (*P* < 0.05) and upper case (*P* < 0.01) letters within a column indicate a significant difference between the various ages of a given plantation type; ns: not significant, *: significant difference (*P* < 0.05), **: extremely significant difference (*P* < 0.01) between the *Eucalyptus* and *Acacia* plantations.

### Total ecosystem biomass and its C stock

The total ecosystem biomass in the two plantations increased sharply with stand age (Table 4). The present data revealed that the ecosystem C stock for the three age classes of *Eucalyptus* plantations was estimated as 29.75, 75.66 and 103.16 t ha^−1^, respectively, while the corresponding estimates for the three age classes of *Acacia* plantations were 43.84, 70.53 and 94.47 t ha^−1^.

## Discussion

### C concentration of the litter layer and the woody debris

The present findings demonstrate that the relative C concentration of both the litter layer and the woody debris in *Eucalyptus* and *Acacia* plantations was species-dependent, partially supporting our first hypothesis. In the present study, a fixed C concentration or mean value, rather than a measured C concentration, would have been inappropriate for calculating the C stock of litter layer and the woody debris for *Eucalyptus* plantations, leading to large estimated error of 12 and 9%, respectively. However, the assumed concentration would have been more accurate for *Acacia* plantations (5% estimated error in the C stock in both studied materials). The implication of this finding is that, in order to accurately estimate the size of the C stock, plantation type-specific measurements are necessary. It has been suggested that the C concentration in the woody debris on the floor of a humid evergreen broad-leaved plantation (49.0%) is very similar to that estimated for the canopy as a whole (50.4%)^24^, consistent with our finding in *Acacia* plantations. A recent study has shown that forest structure, in particular trunk diameter, may influence the C concentration of woody debris^22^. It is likely that the high mortality rate suffered by *Acacia* trees gives other living trees more opportunity to absorb both sunlight and soil nutrient, thereby – at least to some extent - explaining the higher DBH values associated with their arbor layer (Table 1). The observed increase of DBH values implies a rise in the relative contribution of tree trunk wood to the total biomass, which is of relevance given that the trunks’ C concentration tends to be higher than that of other parts of the biomass. As a result, the C concentration of the woody debris in *Acacia* plantations was found to be higher than in the *Eucalyptus* plantations.

The C concentration in the litter layer and woody debris depends on a number of factors, including forest type, stand condition and local climate^6,7,21,24,32–34^. The estimated C concentration of the litter layer in the two types of plantations examined in the present study was comparable with values obtained from the broad-leaved plantations of subtropical China^35^, and local evergreen broad-leaved forests in the PRD region^7,24^. Possibly because the two species stem from the tropical zone, the estimated C concentration of woody debris in the two plantations fell within the range recorded for tropical species^25^. While the estimated C concentration of the woody debris of the *Acacia* plantations was somewhat higher than what has been reported for a tropical mixed *Metrosideros polymorpha/Acacia* plantations^33^, but was comparable with the value obtained from a range of coniferous and broad-leaved forests in temperate climates^21,34^, as well as from evergreen broad-leaved forests in the PRD region^7,36^.

### C stock of the litter layer

The C stock of the litter layer in the two plantations types, particularly the *Acacia*, was compared to estimates derived from the evergreen broad-leaved forests at a nationwide survey^37^, and the broadleaved tree forests in subtropical and mid-subtropical zone^5,38^, specific data see Table 7. The litter layer appears to serve an important role in C accumulation in plantations ecosystem, in particular for *Acacia* plantations. The size of the C stock contained in the litter layer was significantly higher in the *Acacia* than that in *Eucalyptus* plantations, especially in the leaf debris, which was the largest contributor to the litter layer C stock. Its relative contribution to the litter layer reached 72.6% in the *Acacia* plantations, in agreement with both global estimates (60–76%) and its contribution in tropical *Eucalyptus* forests (79%)^32^. In contrast, its contribution in the *Eucalyptus* plantations examined in the present study was only 54.3%. The quantity of leaf litter has been reported to be associated with the annual increment in the DBH^39^. Here, particularly in the *Acacia* plantations, the DBH increased significantly with stand age, implying that the contribution of leaf litter to the C stock would increase accordingly.

**Table 7:** The C stock of the litter layer from the other types of broad-leaved forests (t·ha^−1^).

Item	Forest Type	Stand age (a)	Mean Value (Standard Error)	References and description
Forest floor litter	*Liquidamba formosana*	19	1.88 (0.28)	**References**^5^, subtropical plantations, a total of 18 plots.
	*Schima superb*	19	3.74 (0.23)	
	*Pinus massoniana*	19	4.57 (0.15)	
Litter layer	*evergreen broad-leaved forests at national level*	—	3.21	**References**^37^, 250 sets of data from the reference
	*evergreen broad-leaved forests*	—	4.25~5.57	**References**^38^ 52 stands from young to mature forests in mid-subtropical zone.

Note: — indicates data unavailable or no data.

### Characteristics of the woody debris C stock

#### C stock of woody debris and its distribution in snags and logs

The measured C stock of the woody debris in the two forest types was significantly lower than that reported for both tropical montane wet forests and moist evergreen broad-leaved forests^33,35^. A comparison among evergreen broad-leaved forests in the PRD region revealed that the C stock of woody debris in the *Acacia* plantations was similar to that in forests composed of middle-aged and mature trees reported by Sun and Guan^8^, but significantly lower than that measured in mature forests by Yang *et al*.^2^ It is possible that the high growth rate of *Acacia* does not allow insufficient time for the woody debris to accumulate; the size of the C stock of woody debris in the mature *Acacia* plantations was significantly higher than in the mature *Eucalyptus* ones (6.68 *vs* 1.21 t ha^−1^), levels comparable to those seen in other subtropical plantations^5,40^ (Table 8).

**Table 8:** The C stock in the woody debris from other types of broad-leaved forests (t·ha^−1^).

Item (Diameter)	Forest Type	The given year (stand age)	Mean Value (Standard Error)	References
Wood debris >5 cm	*Liquidamba formosana*	19	7.1 (0.39)	^5,40^
	*Schima superb*	19	0.34 (0.06)	
	*Pinus massoniana*	19	0.45 (0.04)	
	*P. elliottii*	19	0.33 (0.15)	
woody debris (>=10 cm)	Young forests	<40	2.86 (0.48)	^8^
	Middle-aged forests	60–100	5.75 (1.65)	
	Mature forests	>150	11.37 (0.20)	
woody debris (>=10 cm)	Old evergreen broad-leaved forests	1992	7.64	^2^
		1994	9.56	
		1999	11.09	
		2004	11.36	
		2008	19.66	
woody debris (>=2 cm)	Mixed *Metrosideros polymorpha Acacia koa* forests	2009	44.3	^33^
woody debris (>=10 cm)	Primary forests	2007	31.61	^35^

Note: The given year represents the estimated year.

Our study showed that forests type and stand age affect the amount of C stored in woody debris and its distribution in snags and logs. Snags represented the principal component of the woody debris, contributing 60.5% in the middle-aged *Acacia* plantations and 59.0% in the mature ones. These proportions differ markedly from the composition in tropical and subtropical broad-leaved forests, where logs account for 59–95% of the woody debris^2,5,8,40^. It has been suggested that most of the woody debris production (especially snags) is generated as a results of competition between adjacent trees, the death of old trees and external factors, such as weather events, pest and disease damage, and logging^4,33^. A large amount of snags present in *Acacia* plantations may be explained by the high mortality rate of standing trees, in line with conclusions drawn from similar studies^17^. With the exception of the mature *Acacia* plantations, the mortality rate of canopy in our study falls within the range of that for subtropical forests in the PRD region (1.7–12.3% per annual)^41^. The size of the C stock in the logs of the *Acacia* plantations was greater than that for *Eucalyptus*. It is likely that many of these logs were the result of wind-induced breakage; therefore, their frequency is expected to be higher in the *Acacia* than in the *Eucalyptus* plantations.

#### C stock in various diameter class of woody debris

Fallen wood of smaller diameter experiences a greater degree of contact with the ground, and therefore tends to decompose faster^42^. Woody debris material of Small diameter was more frequent in the *Acacia* plantations than in the *Eucalyptus* ones, verifying the hypothesis that forest type affects the C stock within the woody debris of a specific diameter class. The difference between the two plantations types is most likely due to the contrasting wind resistance, particularly for *A. mangium* and *E. urophylla*. The PRD region is the area in China where typhoons occur most frequently, and two strong typhoons were recorded here in 2010 alone. Consequently, the proportion of the C stock within woody debris of small diameter (2.5–10 cm) in the *Acacia* plantations was larger than the range of 40–75% reported in secondary subtropical broad-leaved forests of comparable age^36^, as well as that in forests within the United States (28%)^43^. The C stock proportion in both the mature *Eucalyptus* and *Acacia* plantations (17 and 16%, respectively) was markedly higher than the ~6% reported in primary moist evergreen broad-leaved tropical forests^35^. The C stock contained in the debris of diameter class 10–20 cm represented the largest contributor to the total C stock of woody debris in the middle-age *Eucalyptus* and mature *Acacia* plantations, possibly partially due to the majority of standing trees having a mean DBH of 10–20 cm. This finding further supports the hypothesis that stand age affects the C stock within the woody debris of a particular diameter range.

#### Carbon stock at the stage of various decay class of woody debris

In subtropical natural forests, an estimated 50~90% of the woody debris is in an intermediate-to-advanced state of decay^2,3^; a significantly higher proportion than that observed in the present study. This discrepancy is possibly due to the rapid growth rate of both *Eucalyptus* and *Acacia* trees limiting the time available for woody debris to decay to an advanced degree. Between the two species studies here, the share of the C stock in the woody debris (largely at an early stage of decay) was higher in the *Eucalyptus* than in the *Acacia* plantations. The outcome of the present study verifies the hypothesis that forest type exerts a significant influence on the C stock in the woody debris at particular stages of decay. Even in the mature forests, the C stock contained by material at an early decay stage made a significant contribution to the overall C stock of woody debris. The extent of this contribution was substantially greater than that reported for primary forests (~20%), but markedly lower than that associated with mature evergreen broad-leaved forests and mixed old *Metrosideros polymorpha*/*Acacia koa* forests (64 and 66%, respectively)^2,33^.

In the forest ecosystem, most of the woody debris in the primary decay stage has been created by dead trees and wind damage^3,10^. The snags classed in primary decay in the *Acacia* plantations are likely the result of a combination of typhoons and high mortality rate. A high percentage of woody debris in the plantations of the present study was allocated in decay class I, representing the effect of typhoons. In the *Eucalyptus* plantations, logs were the principal source of woody debris in a primary decay stage, in agreement with conclusions derived from analyses of secondary forests^3^.

### The contribution of the litter layer and woody debris to the ecosystem’s C stock

In terms of the respective contribution of C stock by the litter layer and woody debris to the forest ecosystem, the values determined for the *Acacia* plantations (6.8 and 5.0%, respectively) were significantly higher than those for the *Eucalyptus* plantations (3.7 and 0.7%, respectively). Even in mature *Acacia* plantations the value reached 8.0 and 7.1%, compared with a mere 4.2 and 1.2%, respectively, for mature *Eucalyptus*. These findings indicate that both forest type and stand age impact on the capacity of the litter layer and woody debris to store C, in support of our third hypothesis. The contribution of the litter layer in *Acacia* plantations was higher than that previously reported for subtropical evergreen broad-leaved forests in the PRD region (1.5–6.7%; Table 9), as well as than the estimated worldwide mean of 5%^44^. With respect to the contribution of the woody debris, the value for the *Acacia* plantations was greater than the estimated Guangdong province-wide mean of 3%^9^, but comparable to estimates from local evergreen broad-leaved forests (4.9–6.5%; Table 9). Therefore, both the litter layer and woody debris appear to contribute significantly to long-term C storage, particularly in *Acacia* plantations.

**Table 9:** The C stock of the woody debris and litter layer in subtropical broad-leaved forests (t·ha^−1^).

Ages periods	woody debris/ Litter (mean value (SE))		Ecosystem C stock	Description
	Litter	woody debris		
Young forests	2.12 (0.26)	2.86 (0.48)	5.11	Reference^8^, ecosystem biomass C, author’s calculation
Middle-aged forests	2.45 (0.30)	5.75 (1.65)	116.62	
Mature forests	2.64 (0.31)	11.37 (0.20)	176.23	
Young forests	2.13	1.92	31.96	^40^
Middle-aged forests	3.16	5.52	95.23	
Mature forests	4.37	8.78	194.28	

Note:This was the basic data used for the calculation of the proportional contribution to the ecosystem C stock of the woody debris and the litter layer in subtropical evergreen broad-leaved forests. SE: standard error of the mean; ecosystem biomass C stock: the sum of C stored in the arbor layer, shrub, herb, litter layer and woody debris; diameter of woody debris: ≥10 cm.

### Relationship between total biomass and C stock of litter layer and woody debris

**Figure 1:**
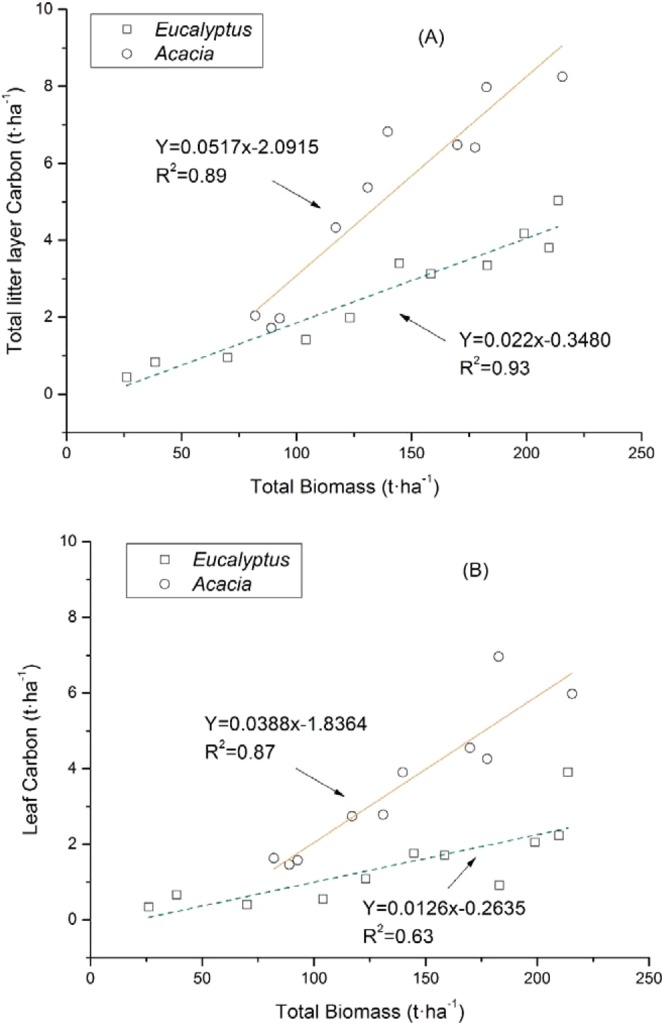
The relationship between the total biomass and the C stock in the litter layer (and its various components) in *Eucalyptus and Acacia* plantations.

**Figure 2:**
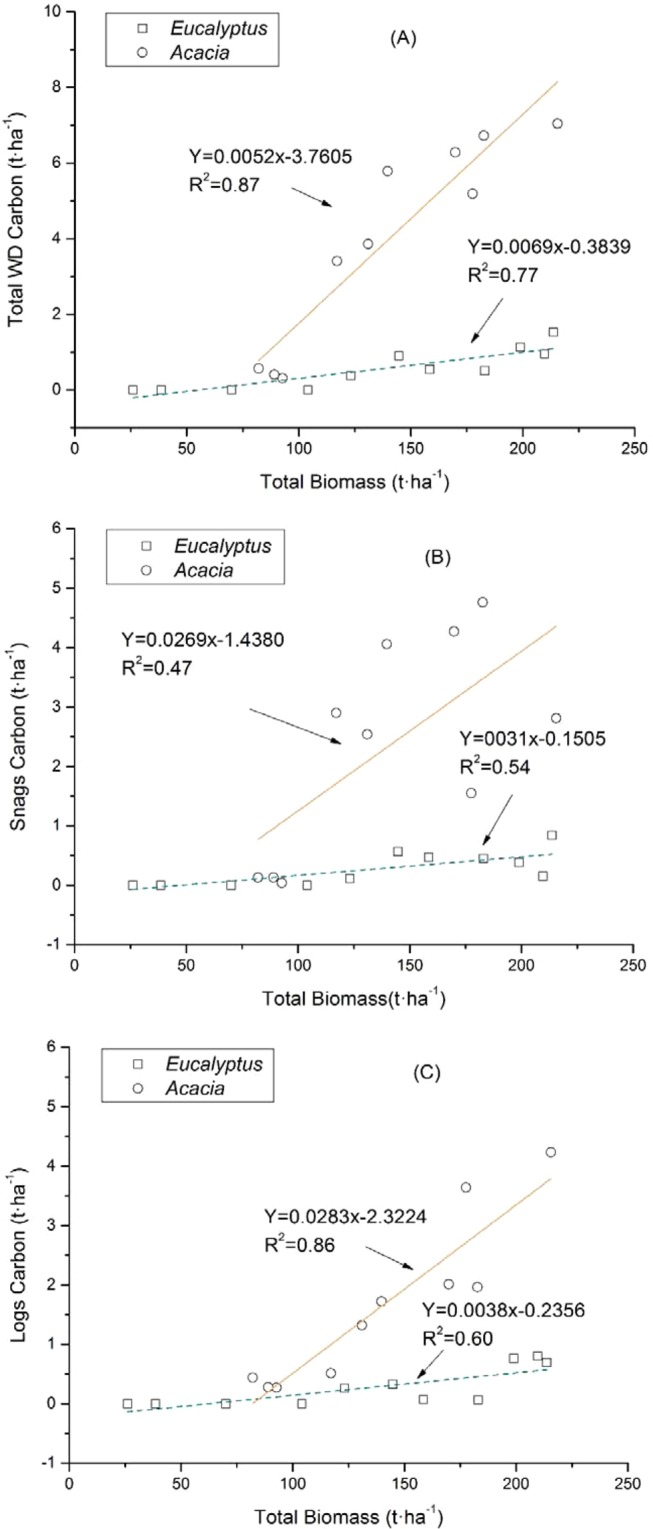
The relationship between the total biomass and the C stock in the woody debris (and its various components) in *Eucalyptus and Acacia* plantations.

The combination of the biomass quantity and the C concentration within it determines the overall size of the C stock present in a given component. A positive correlation was obtained between the total biomass of both the litter layer and the woody debris and their respective components (Figs. 1 and 2), with the highest coefficients of determination corresponding to the total litter layer (0.89 and 0.93 for *Acacia* and *Eucalyptus*, respectively; Fig. 1A) and the total woody debris (0.87 and 0.77 for *Acacia* and *Eucalyptus*, respectively; Fig. 2A). The implication of this finding is that the C stock of the litter layer and woody debris may be restricted by the ecosystem biomass to a great extent. The conclusion is consistent with a study in which the C stock in the litter layer and the woody debris under evergreen broad-leaved trees in southern China was reported to lie in the ranges 2.13–4.37 and 1.92–8.78 t·ha^−1^, respectively, in line with the variation found in the ecosystem biomass (66.3–391.9 t·ha^−1^)^7^.

## Conclusion

The present study describes the characteristics of the C stock in the litter layer and woody debris of *Eucalyptus* and *Acacia* plantations of different ages. *Acacia* trees exhibited a better ability than *Eucalyptus* trees to store C in their litter layer and woody debris; this was especially the case for slags of diameter 10–20 cm in the primary decay stage, which arose from a combination of the natural mortality (especially in the more mature plantations) and natural disturbances such as strong wind. The indications are that forest type as well as stand age are important determinants of the accumulation and distribution of the C stock of both the litter layer and the woody debris. Forest type was also a key driver of the C concentration in both the litter layer and the woody debris, a finding that could improve the accuracy of C stock determination. In order to maximize the C-sequestration potential of the litter layers and woody debris, future plantation management should focus on the tree species selected for reforestation, in particular for N_2_-fixing trees species such as *Acacia*, and on the C-sequestration ability of mature plantations.
